# Editorial: Interactions between diet, sleep and musculoskeletal health: beyond a disease-specific perspective

**DOI:** 10.3389/fnut.2026.1905646

**Published:** 2026-09-02

**Authors:** Xiwei Fan, Yuangang Wu, Antonia Rujia Sun

**Affiliations:** 1 Department of Orthopaedic Surgery, The Second Xiangya Hospital of Central South University, Changsha, Hunan, China; 2 Traumatic Orthopaedic Research Lab, Second Xiangya Hospital of Central South University, Changsha, China; 3 Orthopedic Research Institute and Department of Orthopedics Surgery, West China Hospital, Sichuan University, Chengdu, China; 4 Centre for Biomedical Technologies, Queensland University of Technology, Brisbane, QLD, Australia; 5 School of Mechanical, Medical and Process Engineering, Queensland University of Technology, Brisbane, QLD, Australia

**Keywords:** circadian rhythm, diet, inflammation, musculoskeletal health, sleep

Musculoskeletal health is often discussed through discrete tissues or diagnostic labels: bone loss, cartilage degeneration, tendon injury, muscle wasting and pain. The articles in this Research Topic point to a less tidy reality. Diet quality, the timing of eating, sleep duration and regularity, physical activity, socioeconomic position, mood, endocrine signaling and inflammatory tone may converge on the same tissues, often over many years. This Research Topic therefore looks beyond single-disease framing and asks how diet and sleep are woven into musculoskeletal risk, symptoms and repair across aging, occupational life and chronic disease.

Liu and Xin offer a useful starting point by placing circadian biology at the center of musculoskeletal maintenance. Their review links shift work, sleep restriction and irregular eating patterns with disturbances in clock-gene expression, central-peripheral desynchrony, hormonal imbalance, metabolic dysfunction and chronic low-grade inflammation. These pathways are relevant not only to bone loss, but also to impaired muscle protein synthesis and cartilage degeneration. Liu et al. bring this chronobiological argument into risk prediction by treating night-time eating as a measurable exposure for osteoporosis. Read together, the two papers make a practical point: in aging populations, the timing of food intake may matter alongside nutrient composition, particularly when sleep and eating are no longer aligned with the biological day.

Several contributions move the discussion from bone metabolism to pain, function and repair. In frozen shoulder, Hamed-Hamed et al. report that refined carbohydrates, starch, cholesterol and processed animal products were associated with poorer shoulder function, whereas micronutrient intake, decaffeinated coffee and physical activity were associated with lower pain and disability, especially in women. Law et al. test a related dietary-inflammation hypothesis in symptomatic knee osteoarthritis. Their sex-specific analysis is important: a more pro-inflammatory diet was associated with lower energy in males, but clear associations with sleep, fatigue or psychological distress were not observed. Su et al. add a further caution. In a large UK Biobank cohort, dietary inflammatory potential showed a U-shaped association with fracture non-union, suggesting that repair biology may depend on inflammatory balance rather than simple suppression of inflammation. These studies do not support a blunt nutritional message; they argue for context, tissue specificity and careful separation of association from causation. The review by Na et al. reaches a similar conclusion from an interventional perspective. Exercise showed favorable effects on osteocalcin and appeared protective for lumbar spine and femoral neck bone mineral density, whereas nutritional interventions were less consistent. Polyphenol extracts showed some promise, collagen findings were mixed and micronutrient effects were limited. The review usefully identifies low bone mass as a window for prevention, but it also exposes the weakness of the current evidence base: lifestyle trials remain heterogeneous, often small and still thin in groups that deserve greater attention, including older men.

Muscle health is approached in this Research Topic through both nutrient source and social context. Zhang et al. report that higher intakes of leucine, isoleucine and valine were associated with lower odds of sarcopenia among older adults in Chinese communities, with animal-derived branched-chain amino acids showing a clearer association than plant-derived sources. Liu et al. shift the lens from nutrient classification to lived circumstance. Their finding that the association between tea consumption and appendicular skeletal muscle mass differed by socioeconomic status is a reminder that diet is never only biochemistry; it is also shaped by work, access, culture and social gradients. Lloyd et al. then broaden the nutritional field by testing a cucumber-derived iminosugar supplement and reporting signals consistent with improved sleep quality, urinary melatonin derivatives and finger dexterity in healthy middle-aged and older adults.

Hou et al. do not examine a musculoskeletal endpoint directly, yet their analysis of NHANES 2017-2020 data adds a relevant bridge. By linking sleep disorders, depressive symptoms and gallstone disease, and by positioning depressive symptoms as a partial mediator of sleep-related metabolic risk, the paper shows how sleep, mood and metabolism may cluster within the same behavioral and inflammatory milieu. That milieu is highly relevant to musculoskeletal medicine. Pain sensitivity, fatigue, activity limitation, dietary choice and inflammatory tone rarely remain within neat organ-system boundaries.

Taken together, these articles make the case for an integrated model of musculoskeletal health, but not for a single anti-inflammatory diet or a universal sleep prescription. The more convincing message is that timing, diet quality, physical activity, mood, sex and socioeconomic context should be measured together rather than treated as separate lifestyle variables. The next generation of studies should therefore combine chrononutrition, objective sleep phenotyping, dietary assessment, physical activity measurement and patient-centered musculoskeletal outcomes in longitudinal and interventional designs. Sex and socioeconomic position should be design variables, not afterthoughts. Only then can diet and sleep move from generic lifestyle advice to clinically testable components of musculoskeletal prevention and care.

